# Biological release of phosphorus is more efficient from activated than from aerobic granular sludge

**DOI:** 10.1038/s41598-020-67896-5

**Published:** 2020-07-06

**Authors:** Agnieszka Cydzik-Kwiatkowska, Dawid Nosek

**Affiliations:** Department of Environmental Biotechnology, University of Warmia and Mazury in Olsztyn, Słoneczna 45 G, 10-709 Olsztyn, Poland

**Keywords:** Environmental biotechnology, Environmental sciences, Sustainability

## Abstract

Sewage sludge is a rich source of phosphorus. The kinetics of orthophosphate release and the efficiency of phosphorus recovery from aerobic granular sludge (GS) and activated sludge (AS) were compared at external organics (F) to biomass (M) ratios that ranged from 0 to 0.10. Changes in the F/M ratio affected orthophosphates release from AS to a greater extent than their release from GS. On average, increasing the F/M ratio by 0.02 increased the rate of phosphorus release from AS and GS by 2.12 and 1.75 mg P/(L h), respectively. Phosphorus release was highest at an F/M ratio of 0.04 (114.03 and 60.71 mg P/L from AS and GS, respectively). The efficiency of phosphorus recovery from AS ranged from 51.3 to 56.1%; the efficiency of its recovery from GS ranged from 32.8 to 37.5%. From GS, mostly inorganic phosphorus was released (about 8.5 mg/g MLSS), most of which was NAIP, i.e. phosphorus bound to Fe, Mn and Al. At a stoichiometric dose of MgO to PO_4_^3−^, the precipitation efficiency was 30.13% ± 4.51 with uncontrolled pH and reached 81.73% ± 0.17 at a controlled pH of 10.

## Introduction

Phosphorus is an elementary nutritional component for plants and animals. About 90% of phosphorus in wastewater accumulates in sewage sludge^1^, thus, sewage sludge is perceived as a potential source of phosphorus and various methods of recovering this element in accordance with the assumptions of the circular economy are being developed^2^.

Biological accumulation of phosphorus in excess-sludge biomass is mainly conducted by phosphorus accumulating organisms (PAO) and their activity requires alternating aerobic/anaerobic conditions. In wastewater treatment plants with enhanced biological removal of phosphorus (EBPR), organisms including *Tetrasphaera* sp. and *Accumulibacter* sp. predominate^3,4^. The sludge also contains bacteria that are capable of not only phosphorus accumulation, but also denitrification (DPAO, called Denitrifying Phosphorus-Accumulating Organisms). DPAOs can use nitrates as final electron acceptors for phosphorus uptake under aerobic-anoxic conditions^5,6^.

Phosphorus can be recovered from raw sewage, excess sludge, reject water from sludge dewatering and the ashes formed after sludge combustion. The percent content of phosphorus in dry mass of sludge differs from that in ash after combustion: dewatered sewage sludge contains about 2.6–3.4% of phosphorus^7,8^, whereas the ash after combustion contains 5.9–13.4%^9,10^. Phosphorus in sludge is present in organic and inorganic forms. The main inorganic forms of phosphorus are a fraction adsorbed by exchange sites, called the loosely bound, labile or exchangeable fraction (this is a fraction readily available to plants), an inorganic fraction bound to Ca^2+^ cations and generally referred to as the apatite fraction (AP), which is more bioavailable and can be used directly by plants or in industry^11,12^, and a fraction associated with Al, Fe and Mn oxides and hydroxides, referred to as the non-apatite inorganic phosphorus fraction (NAIP). Pokhrel et al.^13^ observed that total phosphorus in activated sludge comprised 1.7% of the biomass, and consisted of mostly inorganic phosphorus (68–73%), calculated as the sum of NAIP and AP. Li et al.^12^ found that the amount of phosphorus in sewage sludge was 27.03 mg P/g of dried sludge, of which inorganic phosphorus totaled 19.12 mg P/g MLSS, and NAIP accounted for 87% of the inorganic phosphorus.

In anaerobic conditions PAO degrade polyphosphate pool and release orthophosphates to the environment. The obtained energy is used for the uptake of short chain fatty acids that are further stored in cells as polyhydroxyalkanoates. It is possible to increase the rate of phosphorus release from the biomass by adding to the reactor external organics, preferably in the form of acetate^14^, as a food source. The food to microorganisms ratio (F/M ratio) affects not only the release of phosphorus under anaerobic conditions but also biomass growth and pollutant removal^15,16^. Biological phosphorus release from activated sludge increased over 100 times after supplementation with acetate (270 mg/L) in comparison with a sample to which acetate was not added, resulting in release of up to 23% of the total P from the biomass^17^. Addition of acetate at a dose of 1,000 mg COD/L accelerated phosphorus release from the EBPR activated sludge from 1 kg P/m^3^ after 2 days of fermentation to 1.1 kg P/m^3^ after 1 day of fermentation^18^, although it was also observed that the higher acetate dose stimulated cell lysis. Addition of rhamnolipids to activated sludge also caused cell and EPS (Extracellular Polymeric Substances) disintegration, and increased orthophosphate release mostly from strongly bound EPS^19^.

Activated sludge is the most commonly used technology for wastewater treatment all around the world^20^. Activated sludge has a loose structure in the form of flocks, which facilitates the diffusion of nutrients from wastewater to bacterial cells and vice versa, allowing for a high rate of phosphorus release. In recent years, however, there has been increasing interest in aerobic granular sludge technology, which, compared to conventional activated sludge, is an economically and environmentally promising option. Aerobic granular sludge has a compact structure and aerobic, anoxic and anaerobic zones, allowing simultaneous removal of organic nitrogen and phosphorus compounds. Aerobic granular sludge is a promising replacement for activated sludge, for purification of both municipal wastewater and heavily loaded industrial wastewater^21^. Although compact granule structure favors settling and simultaneous nutrient removal, it may affect the rate of phosphorus release, due to the limitation of diffusion of nutrients from the center of the granules.

Despite growing interest in the aerobic granular sludge technology only limited information about phosphorus release is present in the literature and the sludge for the experiments is mostly from laboratory-scale reactors. Compact structure of aerobic granules differs from those of activated sludge which may affect biological phosphorus release from the biomass. Therefore, the aim of this study was to compare the rate and efficiency of the release of orthophosphates from excess activated and aerobic granular sludge obtained from full-scale municipal WWTPs including the predominant type of released phosphorus and possibilities of its recovery due to precipitation in accordance with assumptions of circular economy. To support phosphorus release, external carbon source was used at different F/M ratios.

## Materials and methods

The aerobic granular sludge was taken from the batch reactor of a wastewater treatment plant in Lubawa, and the activated sludge was obtained from an aeration chamber of a wastewater treatment plant in Olsztyn. The treatment plant in Lubawa is operated at a low organics load. The average wastewater flow is approximately 3,200 m^3^/d^22^. Although there is no separate anaerobic phase, at the beginning of the operational cycle there is a feeding phase in which oxygen concentration drops below 0.2 mg/L. Phosphorus content in the biomass was 2.9%. The treatment plant in Olsztyn is a mechanical and biological treatment plant with EPBR. The technological system consists of a pre-denitrification chamber, an anaerobic chamber and a reactor with simultaneous nitrification/denitrification^23^. The average wastewater flow is about 45,000 m^3^/d, and the phosphorus content in the biomass was 3.7%. After sampling, both types of sludge were aerated for 12 h to reduce the orthophosphate concentration in the liquid phase to < 0.5 mg/L.

Activated sludge (5.5 g MLSS/L) was added to six reactors with a capacity of 1 L. Sodium acetate was not added to the control reactor and the ratio of external organics to biomass (F/M ratio) was 0. To the other five reactors, increasing doses of sodium acetate (single dose of 125 mg COD/L) were added resulting in F/M ratios of 0.02, 0.04, 0.06, 0.08 and 0.10. The reactors were sealed with aluminum foil to maintain anaerobic conditions. The experiment was carried out for 125 h until a stable phosphorus concentration in supernatant was reached. Samples were taken from the reactor every several hours to determine the changes in pollutant concentrations. The oxygen concentration in the reactors was below 0.1 mg/L. The experiment was carried out in duplicate. This procedure was repeated for aerobic granular sludge at an identical biomass concentration.

Moreover, at the beginning and end of the experiment, content of particular phosphorus forms in aerobic granules was assessed according to Pokhrel et al.^13^. In short, sludge was incinerated at 450 °C for 3 h, shaken in 3.5 M HCl for 16 h and in the supernatant total phosphorus was measured. Inorganic phosphorus (IP) and organic phosphorus (OP) were assessed by shaking the sludge in 1 M HCl for 16 h. In the supernatant IP was measured while the remaining sludge was incinerated at 450 °C for 3 h and shaken for 16 h in 1 M HCl. In the supernatant OP was measured. NAIP and AP were measured by shaking of sludge in 1 M NaOH for 16 h. The sample was centrifuged. The supernatant was acidified (4 mL of 3.5 M HCl per 10 mL of sample) and left for 16 h at a room temperature, then it was centrifuged and in the supernatant NAIP was measured. The same sludge was once again shaken in 1 M HCl, centrifuged and in the supernatant AP was measured. All analyses were carried out at a room temperature.

Phosphorus precipitation was carried out using MgO (99%, AKTYN). The stoichiometric dose of MgO was calculated based on the concentration of released phosphorus in the supernatant. The stoichiometric and the double dose of MgO were dosed to 500 mL of the supernatant and the mixture was stirred on a magnetic stirrer (WTW OxiTop IS 6-Var) at 250 rpm for 1 h. Precipitation at 8 pH was performed by lowering the pH of the liquid with 2 M HCl just after MgO addition. To obtain 10 and 12 pH, 4 M NaOH was used. The experiment was carried out in duplicate.

COD, orthophosphates and mixed liquor suspended solids (MLSS) were determined according to APHA^24^. Total phosphorus, nitrites and nitrates were determined using the cuvette tests (HACH Lange). The pH was measured with HANNA HI 221 pH meter and the dissolved oxygen concentration was measured with a ProOdo (YSI) probe.

The efficiency of phosphorus release was calculated according to formula 1:

$$\eta = \frac{(Prel-Po)}{Pbiom} \times 100\%$$

P_biom_—concentration of phosphorus in biomass (mg/L). P_rel_—concentration of phosphorus in the supernatant at the end of the experiment (mg/L). P_0_—concentration of phosphorus in the supernatant at the beginning of the experiment (mg/L).

Phosphorus release proceeded according to 1st order kinetics and COD decrease according to pseudo-1st order kinetics.

The results were analyzed using STATISTICA 13.1 (StatSoft) (*p* ≤ 0.05). Averages and standard deviations were calculated. To correlate the results, Pearson coefficient was used (r).

## Results and discussion

In the present study the effect of sludge morphology on the biological release of orthophosphates was investigated. In the activated and granular sludge, the content of phosphorus was 3.7% and 2.9% of the dry mass, respectively (Table 1). These values were similar to those reported for other activated sludge (2.1–2.6%^25,26^) and granular sludge reactors (2.6%^27^). In the presented study, the content of P in the activated sludge was higher than that in the other studies because the system was operated in a manner that favored EPBR (strongly anaerobic chamber in the treatment line). In the sewage treatment plant from which the aerobic granules were collected, there was a feeding phase with quasi-anaerobic conditions; thus, phosphorus accumulation was probably mostly due to the anaerobic zones within the compact structure of the granules.

**Table 1:** Characteristics of the liquid phase and sludge at the beginning of the experiment.

Characteristic (unit)	Activated sludge	Granular sludge
COD (mg/L)	104.0	90.0
Biomass concentration (mg MLSS/L)	5,500	5,500
**TP in the biomass**		
(mg P/g MLSS)	36.9	29.4
(mg P/L)	203.0	161.7
Nitrates (mg/L)	6.3	13.5
Nitrites (mg/L)	0.1	4.9

The main phosphorus fraction in the aerobic granular sludge before the experiment was IP, comprising 75% of the total phosphorus in the sludge. The OP fraction constituted only 25% of the total phosphorus in biomass and was similar to the values reported for activated sludge^13,25^. The AP fraction in aerobic granules accounted for 58% of the IP fraction, which is different from activated sludge because the majority of IP in activated sludge is NAIP^13,25^. The sum of AP and NAIP in granular sludge accounted for about 84.2% of marked IP, which may be associated with the limitations of the of the isolation protocol.

The amount of phosphorus released from the granular sludge in the control reactor was over 10 mg/g MLSS (about 30% of the total phosphorus pool), which is consistent with the phosphorus content in the biomass before and after its release (29.42 and 19.24 mg/g MLSS, respectively). During the experiment, mainly IP was released (around 8.5 mg/g MLSS) and most of this was NAIP, i.e. phosphorus associated with Fe, Mn and Al, which is considered to be unstable and potentially releasable^28,29,30^. After orthophosphate release, NAIP and AP constituted 32% and 68% of the IP remaining in the biomass, respectively. Low AP release may have been associated with pH—the highest AP release was reported at a reaction of about 3 pH^19^. The OP fraction in the biomass after release was slightly lower than before the experiment (a drop from 5.02 to 4.68 mg/g MLSS). The release of phosphorus from the sludge resulted probably from a desorption of phosphorus combined with metals due to a decrease in the redox potential^31^.

With both types of biomass F/M ratio was crucial for the kinetics of orthophosphate release (Table 2), although it did not affect the total amount of orthophosphates released at the end of the experiment from both activated and granular sludge (Supplementary information, Fig. SM1). The phosphorus release was the highest at the F/M ratio of 0.04 (114.03 and 60.71 mg P/L from activated and granular sludge, respectively).

**Table 2:** Kinetics and effectiveness of phosphorus release from activated and granular sludge at various F/M ratios.

F/M	Activated sludge				Granular sludge			
	k (1/h)	C_max_ (mg P/L) (mg P/g MLSS)	r (mgP/(L h))	Ƞ (%)	k (1/h)	C_max_ (mg P/L) (mg P/g MLSS)	r (mg P/(L h))	Ƞ (%)
0.00	0.032	113.0	3.64	55.5	0.035	60.2	2.08	37.2
		(20.5)				(10.9)		
0.02	0.054	106.9	5.75	52.5	0.067	57.4	3.83	35.5
		(19.4)				(10.4)		
0.04	0.289	114.0	32.97	56.1	0.223	60.7	13.52	37.5
		(20.7)				(11.0)		
0.06	0.628	110.2	69.22	54.3	0.298	53.1	15.81	32.8
		(20.0)				(9.6)		
0.08	0.765	106.4	81.34	52.4	0.376	56.1	20.63	33.9
		(19.3)				(10.1)		
0.10	0.847	104.1	88.17	51.3	0.413	55.6	22.96	34.4
		(18.9)				(10.0)		

k, constant of reaction rate; C_max_, maximum concentration; r, reaction rate; ƞ, effectiveness of phosphorus recovery.

In the control reactors, the amount of phosphorus released from the activated sludge (113.0 mg P/L, 20.5 mg/g MLSS) was about 2 times higher than that released from the granular sludge (60.2 mg P/L, 10.9 mg/g MLSS). Taking into consideration the percentage of P in the biomass, 55.5% and 37.5% of phosphorus was released from activated and granular sludge, respectively. The amount of phosphorus released from the granular sludge was about two times higher than that released during fermentation of granular sludge in a study by Zou et al.^27^. In the presented study, analysis of the dynamics of changes in the orthophosphate concentration over time indicated that, if the experiment was carried out for at least 70 h, the final concentrations of orthophosphates in the control reactors and the reactors supplied with the external carbon source were similar. It can be concluded that, if there is enough storage capacity for the sludge, high efficiency of phosphorus release can be obtained without the use of external organics.

**Figure 1:**
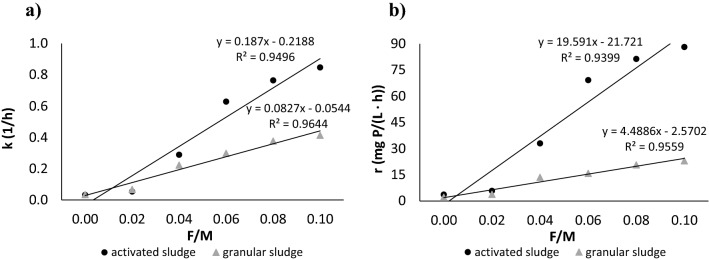
Relationship between the F/M ratio at the beginning of the experiment and (**a**) rate constants for phosphorus release and (**b**) reaction rate of phosphorus release.

The dose of external organics determined, however, the rate constants (Fig. 1a) and reaction rates of phosphorus release (Fig. 1b). The F/M ratio at the beginning of the experiment significantly correlated with the rate constants for phosphorus release from activated (r = 0.98) and granular sludge (r = 0.97) (Fig. 1a). At an F/M ratio of 0.02, the k value for both types of sludge was almost twice as large as that for control (Table 2). At an F/M ratio of 0.04, the rate constants for activated and granular sludge were more than ninefold and sixfold higher, respectively, than that for the control reactors (Table 2).

In the control reactors, the COD concentration increased by about 350 and 200 mg/L at the end of the experiments with activated and granular sludge, respectively, which may be related to the release of carbon compounds during cell lysis. This lysis was less visible for aerobic granules which are regarded as resistant to EPS decomposition in famine periods^32,33^. The concentration of COD decreased in the first 3 h of the experiment in all reactors to which sodium acetate was dosed at rates of 52.83 to 68.57 mg COD/(L h) for activated sludge and 43.26 to 72.86 mg COD/(L h) for granular sludge (data not shown). With activated sludge, the largest decrease in COD concentration (330 mg COD/L to 160 mg COD/L) was observed at the F/M ratio of 0.06; with granular sludge, the largest decrease (387 mg COD/L to 186 mg COD/L) was observed at the F/M ratio of 0.08.

**Figure 2:**
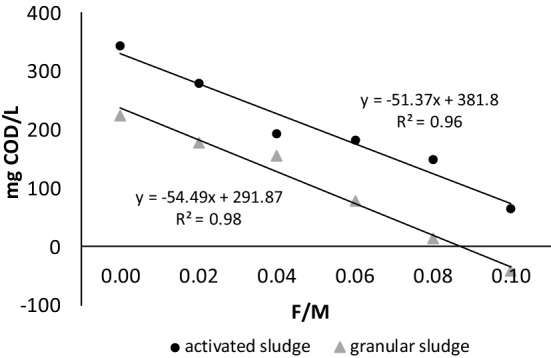
Relationship between the change in COD concentration during the experiment and the F/M ratio at the beginning of the experiment; values on the vertical axis correspond to the increase or decrease in COD concentration during the experiment.

In the reactors supplied with external organics, COD concentrations increased from the 4th hour until the end of the experiments at rates from 2.1 to 2.8 mg COD/(L h) in activated sludge and from 1.1 to 2.0 mg COD/(L h) in granular sludge reactors. These increases in COD concentration at the end of the experiment indicated that microorganisms consumed more dissolved COD as the F/M ratio was increased (Fig. 2). When digesting sludge from an EBPR treatment plant, Wang et al.^18^ observed that the concentration of organic matter and the fraction of living cells in biomass decreased as the initial concentration of external carbon source was increased. Those authors pointed out that an increased content of polyhydroxyalkanoates accelerates cell lysis, which increases the amount of dissolved COD. In all reactors in the presented study, the biomass concentration decreased during the experiments. With both types of biomass, this decrease was highest (16.5%) at an F/M ratio of 0.04. The release of phosphorus was also highest at this ratio, which indicates that some of the carbon released as a result of lysis may have been used for the release of orthophosphates.

The effectiveness of total phosphorus release from granular sludge depends on pH. Without pH regulation the recovery was at a level of 17%, but at pH 4, it increased to about 80% after 144 h of fermentation^27^. In the presented study, pH was not regulated to limit the amount of chemicals used in the process. At the beginning of the experiments, the pH of the supernatant liquid was similar in both activated and granular sludge reactors (Supplementary Materials Fig. SM2). With both types of biomass, the pH decreased over time, and the rate was highest between the 3rd and 20th hour of the experiment. During this time, the rate of increase of the orthophosphate concentration in the supernatant was also highest. At the end of the experiments, the pH in the reactors with activated sludge ranged from 6.8 to 7.1, while in the reactors with granular sludge it ranged from 7.1 to 7.5. Under lower reactions, phosphorus can be released in larger amounts as a result of dissolution of inorganic phosphorus^26^. For example, Wu et al.^34^ used initial sludge acidification to increase phosphorus release, and after 84 h of the experiment, the concentration of orthophosphates increased from 28.2 mg/L to about 100 mg/L, which is close to the maximum values obtained for activated sludge in the presented study.

Activated sludge flocs have a loose, irregular structure, which allows the substrates and the products of microbial metabolism to easily diffuse from the liquid phase to the microorganisms or vice versa. In contrast, aerobic granules have a compact, multi-layered structure. Although there are also numerous channels and pores in the granules, the transport of nutrients to the interior of the granule is limited. In the inorganic core of the granule, phosphorus compounds are precipitated and it is difficult or impossible to release them without granule disintegration. The efficiency of phosphorus recovery from activated sludge ranged from 51.3 to 56.1%, whereas the efficiency of its recovery from granular sludge ranged from 32.8 to 37.5% (Table 2). Overall, the efficiency was highest at an F/M ratio of 0.04. In the presented study, the amount of biological phosphorus in both types of biomass was similar, so the observed differences in phosphorus release were likely due to biomass morphology.

### Phosphorus precipitation

**Figure 3:**
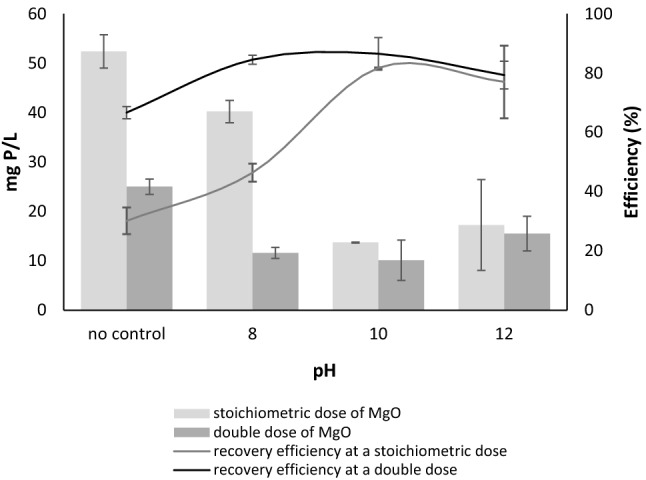
Phosphorus concentration in a supernatant after precipitation with a stoichiometric and a double dose of MgO and the efficiency of phosphorus recovery.

Finally the possibility of phosphorus recovery by chemical precipitation was tested. Figure 3 presents the efficiency of phosphorus recovery and the concentration of orthophosphates in the supernatant after precipitation with two MgO doses at different reactions (uncontrolled reaction and at reaction of 8, 10 and 12 pH).

Studies have shown that pH affected orthophosphate precipitation at the stoichiometric dose of MgO. The precipitation efficiency was 30.13% ± 4.51 and 46.37% ± 3.03 for the uncontrolled pH and 8 pH, respectively. There was no statistical difference between efficiency of precipitation at 10 and 12 pH (precipitation efficiencies were 81.73% ± 0.17 and 77.02% ± 12.28, respectively). At a double dose of MgO, the lowest efficiency of phosphorus precipitation was obtained at the uncontrolled pH (66.59% ± 2.08), at reactions from 8 to 12 pH, this efficiency varied between 79 and 85%. Studies by Li et al. ^35^ indicate that 10 pH promotes phosphorus precipitation with magnesium ions with an efficiency of 90–98% at a Mg/P ratio of 1–1.8. However, the mere addition of Mg, without raising the pH, resulted in a very low recovery efficiency of phosphorus precipitation (about 50% at Mg/P ratio of 1.8). In addition, Fourier Transform Infrared Spectroscopy showed that phosphorus precipitated by magnesium ions at 10 pH elicited affinity for struvite^35^. Studies by Stolzenburg et al.^36^ show that precipitation of struvite by MgO occurs faster and more efficiently (90% efficiency at Mg/P ratio of 1.0) than with MgCl_2_.

The precipitation experiment showed that at uncontrolled pH and pH of 8, the dose of MgO significantly affected phosphorus recovery, however, at higher reactions the phosphorus recovery was similar independent of the MgO dose.

## Conclusions

In the present study, recovery of phosphorus from activated and granular sludge obtained from full-scale facilities with a long history of operation was compared. Such data are especially important for obtaining information about the costs and benefits of implementation of aerobic granular sludge. The study showed that the biomass morphology has a substantial effect on phosphorus release. Despite the similar content of biological phosphorus in both types of biomass, the efficiency of orthophosphates recovery from activated sludge ranged from 51.3 to 56.1%, whereas recovery from granular sludge ranged from 32.8 to 37.5%. Dosing of external organics (F/M ratio from 0.02 to 0.08) increased the rate of phosphorus release from both types of biomass, but the reaction rates were up to 4.5 times higher with activated sludge than with granules. From aerobic granules, mostly the IP fraction was released. The results of this study indicate that the best option is to store an excess sludge in a thickener for about 70 h and then precipitate phosphorus from the supernatant with a dose of MgO two-times higher that stoichiometric to obtain the efficiency of phosphorus recovery of 79 to 85%.

## Supplementary information

Supplementary Figures
