# Supplementary material for: Biological release of phosphorus is more efficient from activated than from aerobic granular sludge
Source: Sci Rep. 2020 Jul 6;10:11076. doi: 10.1038/s41598-020-67896-5 (PMC7338368; doi:10.1038/s41598-020-67896-5)
Supplement: Supplementary file 1 — Supplementary Figures [file 41598_2020_67896_MOESM1_ESM.docx]

**Supplementary materials**

**Biological release of phosphorus is more efficient from activated than from aerobic granular sludge**

Agnieszka Cydzik-Kwiatkowska, Dawid Nosek*

University of Warmia and Mazury in Olsztyn, Department of Environmental Biotechnology, 10-709 Olsztyn, Słoneczna 45 G

Corresponding author: dawid.nosek@uwm.edu.pl, tel. +48 89 5234144

**Figure SM1** Relationship between the final concentration of orthophosphate released and the F/M ratio at the beginning of the experiment

**a)**

**b)**

**Figure SM2** Changes of pH during the release of orthophosphates in a) activated sludge and b) granular sludge reactors.
